# FlaME: Flash Molecular Editor - a 2D structure input tool for the web

**DOI:** 10.1186/1758-2946-3-6

**Published:** 2011-02-01

**Authors:** Pavel Dallakian, Norbert Haider

**Affiliations:** 1 Department of Drug and Natural Product Synthesis, University of Vienna, Althanstraße 14, A-1090 Vienna, Austria

## Abstract

**Background:**

So far, there have been no Flash-based web tools available for chemical structure input. The authors herein present a feasibility study, aiming at the development of a compact and easy-to-use 2D structure editor, using Adobe's Flash technology and its programming language, ActionScript. As a reference model application from the Java world, we selected the Java Molecular Editor (JME). In this feasibility study, we made an attempt to realize a subset of JME's functionality in the Flash Molecular Editor (FlaME) utility. These basic capabilities are: structure input, editing and depiction of single molecules, data import and export in molfile format.

**Implementation:**

The result of molecular diagram sketching in FlaME is accessible in V2000 molfile format. By integrating the molecular editor into a web page, its communication with the HTML elements on this page is established using the two JavaScript functions, *getMol() *and *setMol()*. In addition, structures can be copied to the *system clipboard*.

**Conclusion:**

A first attempt was made to create a compact single-file application for 2D molecular structure input/editing on the web, based on Flash technology. With the application examples presented in this article, it could be demonstrated that the Flash methods are principally well-suited to provide the requisite communication between the Flash object (application) and the HTML elements on a web page, using JavaScript functions.

## Background

### About the Project

At present, there are various tools available for structure input on web pages, for example Marvin Sketch [1], PubChem Sketcher [2], NIST Molecule Editor [3] or JME [4]. The latter one is very popular because of its compact footprint and intuitive use. It has been utilized by many web developers (including the authors of this article) for sites with chemical content. Recently, a comprehensive review article [5] summarized the developments in this field. Prompted by the statement that so far no Flash-based web tool for structure input is available, we decided to undertake a feasibility study, aiming at the development of a compact and easy-to-use 2D structure editor, using Adobe's Flash technology and its programming language, ActionScript. As a reference model application from the Java world, we selected the Java Molecular Editor (JME). In this feasibility study, we made an attempt to realize a subset of JME's functionality in the FlaME utility (*part I, this article*). These basic capabilities are: structure input, editing and depiction of single molecules, data import and export in molfile format. As this is just the beginning of the project, we intend to continue and extend this work with additional features like reaction editing and the implementation of other data exchange formats (e.g., SMILES) (*part II, in planning*).

### Objectives and expectations

The primary objective of this work has been to demonstrate a usable Flash application (written in Flash/ActionScript) for drawing and editing molecules directly within a HTML/JavaScript web page. The goals are as follows: a) we want to achieve the convenience of common structure editors such as ISIS (Symyx) Draw [6] or Marvin Sketch [1], which are easy and intuitive to use; b) at the same time the application should be compact - in other words: small and smart. As the data exchange format (*result or output format*), the well-documented and proven MDL MOL format V2000 [7] was chosen. This format is still very popular for structural data exchange and it is used by many databases and cheminformatics applications, despite its inherent limitations, e.g. with respect to the maximum number of atoms. The latter aspect, however, does not affect its suitability for FlaME, as this editor is primarily designed to handle "small molecules".

## Results and discussion

### The FlaME Molecular Editor: features - graphical user interface and layout

**Figure 1:**
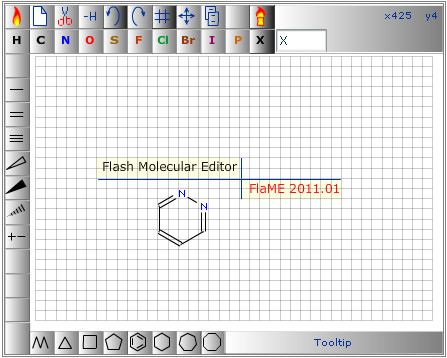
**FlaME graphical user interface**.

As mentioned above, the authors were inspired by the JME applet written by P. Ertl [4] when designing the layout of the graphical user interface (GUI). Nevertheless, we tried to create a typical and unique look-and-feel for our new tool (Figure 1). Actually, there should be no explanation needed for a novice user of FlaME. Users should immediately feel familiar with the buttons and functions in FlaME. Furthermore, tooltips are displayed in the right bottom area of the GUI which are activated by slowly moving the mouse pointer over the various buttons, thus facilitating use of the applet.

### GUI buttons

**Figure 2:**
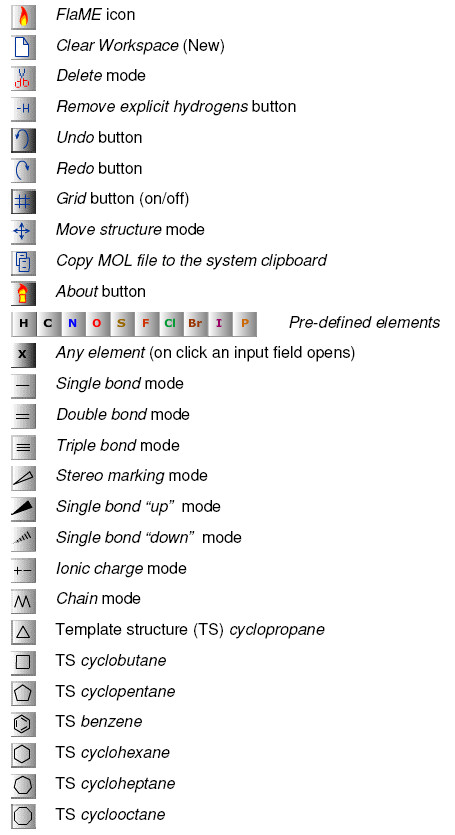
**Description of the GUI buttons**.

The FlaME GUI is simple and all icons on the buttons should be intuitively understood. Figure 2 shows the icons and a description of the individual buttons.

### Subjects and actions

Based on the definition of operations (drawing primitives) for molecular structure diagram sketching as discussed by A. M. Clark [8], we can define the subjects in FlaME as atoms and bonds. The actions implemented so far are as follows:

- add or delete atom

- add or delete bond

- add structure fragment (as pre-defined *template structure*)

- set or change the atom type (as a pre-defined element or as input via X-button)

- set or change the bond order

- set or change the stereo style (*stereo marking*)

- set or change the ionic charge

The element or group label will change automatically depending on element and current environment of the particular structure node (*node environment*).

### Molecular structure output

The result of molecular diagram sketching in FlaME is accessible in V2000 molfile format [7]. This result can be copied to the *system clipboard *(by clicking the corresponding button). On the other hand, by integrating the molecular editor into a web page, its communication with the HTML elements on this page must be established. For this purpose, two JavaScript functions are implemented which are described below.

### Flash Methods - JavaScript getMol() and setMol() functions

The Flash documentation states: "*A Flash method is a JavaScript function that is specific to Flash movies. Use Flash methods to send JavaScript calls to Flash movies from a scripting environment*." [9]

Using the Flash methods, two functions (*getMol *and *setMol*) were written: the function *getMol() *ensures that the structure drawn in the molecular editor will be copied as a molfile to a *textarea *field (*id = "mol"*) within the web page for further use, e.g. as a query structure for a database search. The second JavaScript function *setMol() *sends the molfile from the web page to the Flash object, in our case to the FlaME molecular editor. The second line in this function just ensures compatibility with all common web browsers, such as Internet Explorer, Firefox, Opera, Chrome, Safari etc.

While the purpose of the *getMol() *function could be described as "*export as MOL file*", the *setMol() *function could be called "*import MOL file*" and it can be used for structure input in textual form for further structural modification using FlaME (see example 1).

function getMol() {

var m = thisMovie("flame").GetVariable("mol");

document.getElementById("mol").value = m;

}

function setMol() {

var m = document.getElementById("mol").value;

m = m.replace(/\r\n/g, "\r");

thisMovie("flame").send2Flame(m);

}

### Drawing operations and template structures

The actions of FlaME implemented so far do not reflect all of the basic primitives for molecular diagram sketching, described previously [8]. It was not the purpose of this tool to cover all these possible functionalities, but to provide users (and developers) with an opportunity to use a rather compact input tool for chemistry-related websites. The standard template structures included in FlaME are: cyclopropane, cyclobutane, cyclopentane, cyclohexane, benzene, cycloheptane, and cyclooctane. Once placed in the drawing area, these structures can be edited by changing the bond order (single, double and triple), by changing the atom type (element), or by changing the stereo style (stereo marking).

### Use of a special key

**Figure 3:**
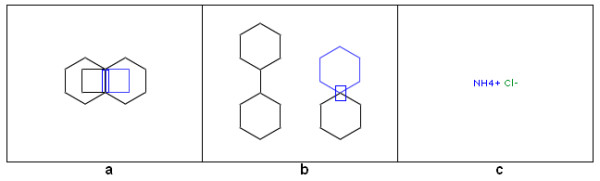
**Using the Shift key while editing a molecular structure**.

The *Shift *key on the computer keyboard can be used to modify some of the drawing operations of FlaME: whereas ring fusion (annulation) by clicking with a ring template on an existing ring bond normally produces a fully expanded condensed system, pressing the *Shift *key while clicking will cause the new ring to be flipped to the opposite direction (Figure 3, structure a). Similarly, adding a ring template to an existing ring atom normally produces two rings connected by a single bond, whereas the *Shift *key modifies this behavior and leads to a spiro ring system (Figure 3, structure b). Furthermore, by pressing the *Shift *key the user can prevent the delete operation from removing terminal ("orphan") atoms when a bond is deleted (Figure 3, structure c). The latter option can be used to create disconnected structures (e.g., salts) from a single molecule, thus providing a work-around for the current limitation of FlaME to handle only a single connection table.

### Features which are not yet implemented

Compared to the JME applet, FlaME still lacks a number of features which may be implemented in future versions. For instance, there is currently support for only one connection table at a time. Thus, there is no mode available for reaction input and depiction. Special definitions for query atoms are limited to A, Q, and X (see below), while R groups are not yet supported. Moreover, at present the only data exchange format is the MDL molfile format (V2000), whereas SMILES export is still on the to-do list.

## Implementation

### Development environment

Flash applications are frequently used for advertisements and as games. Very useful applications have been also developed and widely employed for e-learning purposes. Flash as well as Java and JavaScript can be used to add interactivity to web pages using the programming language ActionScriipt, which has a similar syntax and semantics as JavaScript (or, more generally, is a dialect of ECMAScript). The FlaME application itself was developed using Adobe Flash Professional CS4, which provides an integrated development environment. It is equipped with a compiler which can produce either compact SWF files suitable for use on web pages, or so-called Windows-projectors or Macintosh-projectors which are platform-specific executables.

FlaME is a single-file Flash application. It was written in ActionScript (AS2) and compiled as a Flash 8 SWF file. Thus, this application requires a common web browser (Firefox, IE, Opera, Chrome, Safari etc.) and the Flash Player plug-in at least in version 8. According to Adobe: "*Today, over 86% of Internet-connected computers have adopted the Flash Player version 9*" [10].

Whereas there should be no principal (technical) problem to make use of an Adobe Flash Player also on Apple's mobile devices (iPhone, iPad and iPod touch), Apple has decided not to support Flash on these devices and therefore, applications such as FlaME cannot be used on them. However, essentially the same restriction applies to Java on the iPhone/iPad which also prevents Java-based structure editors from being employed on these mobile devices. Although there are some "inofficial" ways to install unsupported software on Apple's mobile devices, only a change in Apple's policy would lead to a general solution of this Flash/Java lack-of-support issue. It should be noted, however, that FlaME was developed for use on typical desktop computers rather than on mobile gadgets.

### Limitations

Because of the inherent limitations of the V2000 MDL molfile format (see the CTfile formats description [7]) which is used by FlaME for data exchange, the maximum number of atoms is limited to 999.

### FlaME application examples

In this section, three application examples are presented which have been built around FlaME and which are freely accessible on the web. A common web development practice is to use both an <object> tag and an <embed> tag to display Flash (SWF) content within an HTML page [11]. The nested-objects method requires a double *object *definition (the <object> tag targeting older versions of Internet Explorer and the <embed> tag targeting all other browsers), so it is necessary to define the object attributes and nested *param *elements twice [12]. The required attributes are: *classid *(the value is always as shown below), *type*, *width*, and *height*. The required *param *element is *movie*, which defines once more the URL of a SWF file. For up-to-date web browsers, one specifies the URL of the Flash application with the *src *attribute.

### Embedding SWF content (i.e., the FlaME application) in HTML

<object classid='clsid:d27cdb6e-ae6d-11cf-96b8-444553540000'

id='flame' height='438' width='350' align='middle'>

<param name='allowScriptAccess' value='sameDomain'/>

<param name='movie' value='flame.swf'/>

<param name='quality' value='high'/>

<param name='bgcolor' value='white'/>

<embed src='flame.swf' quality='high' bgcolor='#ffffff' width='438' height='350'

name='flame' align='middle' allowScriptAccess='sameDomain'

type='application/x-shockwave-flash'

pluginspage=http://www.macromedia.com/go/getflashplayer/>

</object>

### Application example 1: Structure Search

**Figure 4:**
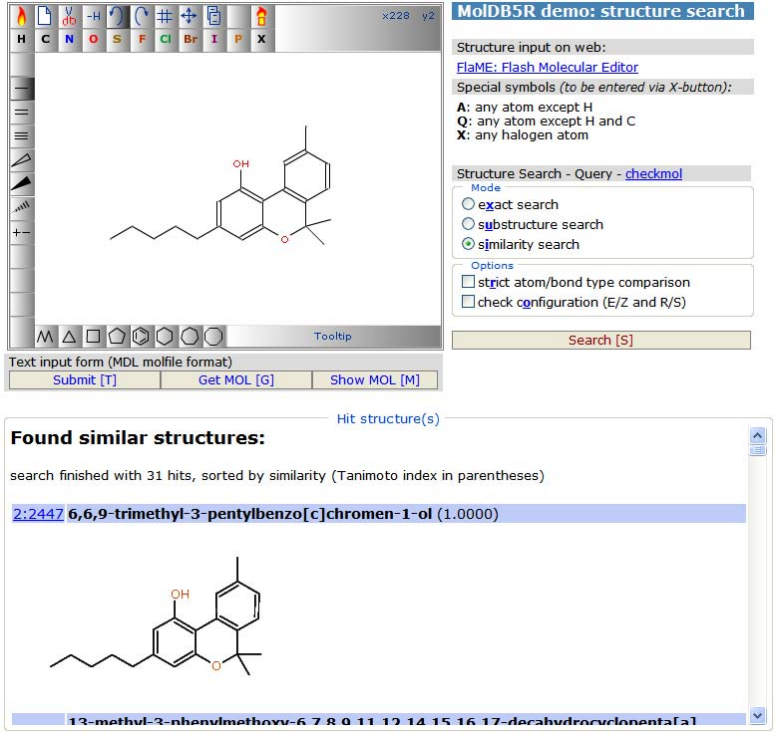
**MolDB5R similarity search using FlaME for query input**.

**Figure 5:**
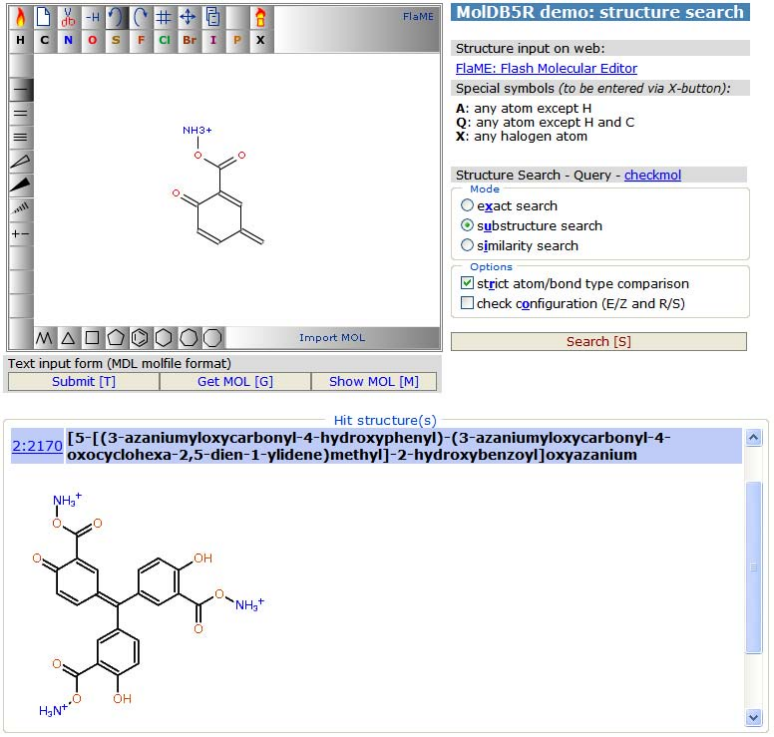
**MolDB5R substructure search using FlaME for query input**.

This demonstration page [13] uses FlaME as a replacement for JME in a modified version of the open-source structure database system MolDB5R [14-16]. The latter software employs the *checkmol/matchmol *program [17] as the search engine. Figure 4 shows a run in "similarity search" mode (using the Tanimoto index [18] derived from binary fingerprints as similarity criterion), Figure 5 shows another example in "substructure search" mode. Like with JME, special symbols (to be entered via the X-button) can be used for the definition of query atoms:

**A **for any atom except H

**Q **for any atom except H and C

**X **for any halogen atom

Explicit **H**ydrogens are entered via a separate H button.

### Application example 2: Presentation of structure collections in "slide show" mode

**Figure 6:**
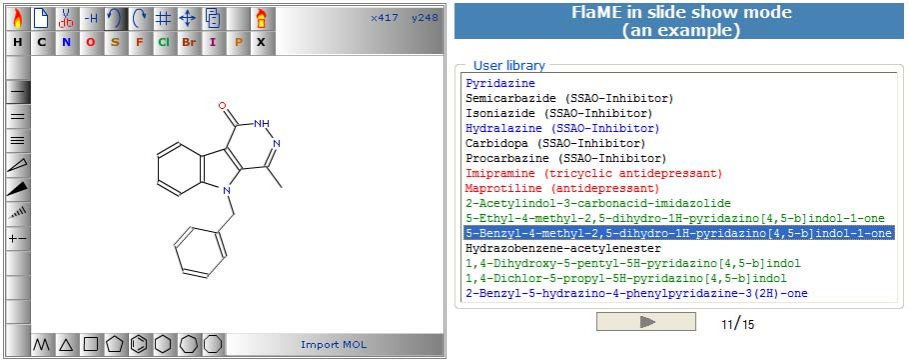
**FlaME demo page in "slide show" mode**.

This web page (Figure 6) uses the JavaScript function *setMol() *to send the molfile of the selected structure to FlaME. The user can either send any of the listed structures to FlaME by clicking on the respective chemical name in the right area (printed in arbitrary color) or he/she can start a "slide show" by selecting the "play" button below the list. In the latter case, the data transfer process runs automatically with a pre-defined time interval.

### Application example 3: FlaME editor vs. depiction

**Figure 7:**
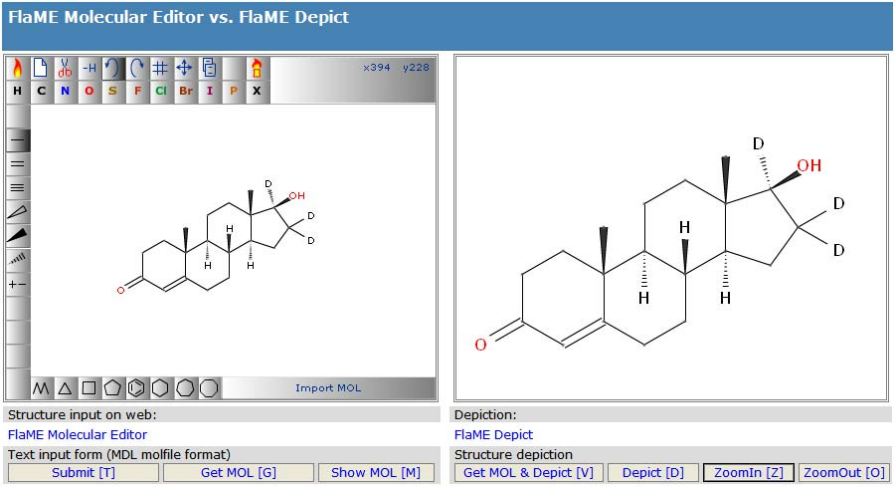
**FlaME: Molecular Editor vs. Depiction**.

This example (Figure 7) shows the FlaME editor (left side) in combination with a depiction-only version of the software (right side). In the latter incarnation, the "move structure" mode is activated by default, as well as "zoom in" and "zoom out" functions which are accessible on a web page via keyboard shortcuts or via HTML elements (buttons) connected to appropriate JavaScript functions.

This depiction-only version of FlaME was compiled separately without any GUI buttons and layout options. By doing so, the size of the SWF file could be reduced to approximately one third (25 kB) of the original file size of the editor version.

## Conclusions and Outlook

A first attempt was made to create a compact single-file application for 2D molecular structure input/editing on the web, based on Flash technology. At present, the project should be regarded as a feasibility study and any feedback (comments and suggestions for further development and extensions) will be welcome.

With the introduction of the Flash Molecular Editor (FlaME) as an alternative to similar input tools based on Java technology, we would like to start a discussion about the suitability (advantages and limitations) of the Flash environment for interactive chemistry-related websites. With the application examples presented in this article, it could be demonstrated that the Flash methods are principally well-suited to provide the requisite communication between the Flash object (application) and the HTML elements on the web page, using JavaScript functions.

The next version of FlaME should include a reaction editor and some other extensions, for instance generation of SMILES [19] output. For future developments, we envisage a separate Flash application for handling spectral data, e.g. for JCAMP-DX spectra viewing/editing. In this field, there are already some nice plug-ins and Java applets available (e.g., MDL Chime [20], JSpecView Applet [21], or the NIST JCAMP-DX Viewer [22]), which could be taken as models for writing a comparable Flash-based tool.

### Use and Reference

Developers of chemistry-enabled websites are invited to try FlaME on their pages. The utility [Additional file 1] can be obtained free of charge from the authors. As the only condition for its use, we ask to place an appropriate reference next to the applet. The authors provide the present version of FlaME only as compiled SWF file, but we are contemplating the possibility to make it an open-source project in the future. We are aware of the fact that the major Flash development tools are not free. For a true open-source project, an open-source development tool such as AJAX Animator [23] would be desirable. However, at present the latter product is still far away from its goal to be a complete web-based animation suite.

## Availability and requirements

Project name: FlaME: Flash Molecular Editor - a 2D structure input tool for the web

Project homepage and application examples URL: http://synthon.pch.univie.ac.at/flame/

Application download URL: http://synthon.pch.univie.ac.at/flame/flame.swf

Operating system(s): Platform independent

Programming language: Flash, ActionScript 2

Other requirements: Adobe Flash Player 8 or higher

License: individual license on request (free of charge).

Any restrictions to use by non-academics: none

## Competing interests

The authors declare that they have no competing interests.

## Authors' contributions

PD was involved in the design and programming of the FlaME software, in the implementation of the software and preparation of the application examples, and the manuscript preparation. NH was involved in testing and intellectual guidance during FlaME software development, in preparation of application examples, and manuscript preparation. All authors read and approved the final manuscript.

## Supplementary Material

Additional file 1 **FlaME - Flash Molecular Editor**. Platform independent SWF application file Click here for file
